# Polyimide-Based Capacitive Humidity Sensor

**DOI:** 10.3390/s18051516

**Published:** 2018-05-11

**Authors:** Jamila Boudaden, Matthias Steinmaßl, Hanns-Erik Endres, Andreas Drost, Ignaz Eisele, Christoph Kutter, Peter Müller-Buschbaum

**Affiliations:** 1 Fraunhofer EMFT, Research Institution for Microsystems and Solid State Technologies EMFT, Hansastraße 27d, D-80686 Munich, Germany; matthias.steinmassl@emft.fraunhofer.de (M.S.); hanns-erik.endres@emft.fraunhofer.de (H.-E.E.); andreas.drost@emft.fraunhofer.de (A.D.); ignaz.eisele@emft.fraunhofer.de (I.E.); christoph.kutter@emft.fraunhofer.de (C.K.); 2 Institute of Electronic and Sensor Materials, Technische Universität Bergakademie Freiberg, Gustav-Zeuner-Str. 3, D-09599 Freiberg, Germany; 3 Physik-Department, Lehrstuhl für Funktionelle Materialien, Technische Universität München, James-Franck-Strasse 1, D-85748 Garching, Germany; muellerb@ph.tum.de; 4 Physik-Department, Universität der Bundeswehr München, Werner-Heisenberg-Weg 39, D-85579 Neubiberg, Germany

**Keywords:** humidity sensor, flat polyimide, nanograss polyimide, rough polyimide

## Abstract

The development of humidity sensors with simple transduction principles attracts considerable interest by both scientific researchers and industrial companies. Capacitive humidity sensors, based on polyimide sensing material with different thickness and surface morphologies, are prepared. The surface morphology of the sensing layer is varied from flat to rough and then to nanostructure called nanograss by using an oxygen plasma etch process. The relative humidity (RH) sensor selectively responds to the presence of water vapor by a capacitance change. The interaction between polyimide and water molecules is studied by FTIR spectroscopy. The complete characterization of the prepared capacitive humidity sensor performance is realized using a gas mixing setup and an evaluation kit. A linear correlation is found between the measured capacitance and the RH level in the range of 5 to 85%. The morphology of the humidity sensing layer is revealed as an important parameter influencing the sensor performance. It is proved that a nanograss-like structure is the most effective for detecting RH, due to its rapid response and recovery times, which are comparable to or even better than the ones of commercial polymer-based sensors. This work demonstrates the readiness of the developed RH sensor technology for industrialization.

## 1. Introduction

Humidity is defined as the water vapor amount in air. It is the most abundant greenhouse gas and an important abiotic factor influencing the human life. To describe humidity in a precise way, several items are commonly used, which are absolute humidity (AH), relative humidity (RH), specific humidity (SH), and dew point [1]. The RH term is commonly employed to describe the content of water vapor in the surrounding atmosphere by taking into consideration both temperature and pressure. 

Recently, the state of the art of humidity monitoring has evolved rapidly and continuously. However, the wide application range of devices being able to record humidity is behind the intensive scientific studies dedicated to this area. Their most important fields of use are indoor application, plants growth in agricultural market, and control of industrial processes, electronics, and semiconductor area, as well as medical processes [2,3,4,5,6,7,8].

A survey of the literature leads to a classification of humidity sensors based on their working principle as capacitive, electrical conductivity (or resistive) and optical sensors. It is obvious that, except optical sensors, a humidity sensor is defined as a combination of a transducer and a sensing material to humidity.

Interdigitated transducers (IDT), also known as interdigital electrodes, have been intensively studied and used for unlimited applications due to the possibility of measuring both capacitance and resistance [9,10]. An IDT is a structure consisting of two comb-like electrodes on a planar substrate. Their technological realization is readily mastered in the semiconductor field. To design a relative humidity sensor by considering the IDTs as transducer, three main sensing materials were often reported by the scientific community: ceramic, semiconductor, and polymer materials [7].

Ceramic materials, like Al_2_O_3_, TiO_2_, SiO_2_, and spinel compounds with AB_2_O_4_ structural composition, are known to change their electrical properties, such as resistance, in the presence of humidity [7]. The first initial adsorbed layer of water molecules is stable and ordered. A chemical reaction between water molecules and the metal oxide surface was suggested to form two hydroxyls group [11]. Each single water molecule is attached to two neighboring hydroxyls by hydrogen bonding [12], thus a second water layer is formed. By further adsorption of water vapor, additional layers of water molecules bond by weak forces (van der Waals-type) in a disordered manner on the surface of the first physically adsorbed layer. Hence, proton conduction takes place from one oxygen atom (in a water molecule) to the next one, through the Grotthus mechanism [13]. This mechanism requires disorder, which is not obtained during the formation of the first chemisorbed and the first physisorbed layers. Therefore, the conductivity mechanism starts upon the second physisorbed layer. Consequently, at low humidity, the conductivity is almost unchanged. For this reason, most ceramic materials are not sensitive to RH below 20% [14]. Decreasing the ceramic’s pore size enhances the sensitivity at lower humidity level. Unfortunately, this decrease goes ahead with a faster degradation due to clogged small pores with dirt.

Semiconducting materials, like SnO_2_, In_2_O_3_ [15], and perovskites [16], are promising candidates for sensing humidity with a high precision. Existing research activities focus mostly on n-type semiconductors, for which the conductivity increases with increasing humidity. Semiconductors show physical adsorption of water, where two mechanisms contribute to the change in its electrical conductivity. The adsorption happens at dangling bonds of the semiconductor surface molecules or by van der Waals interaction to surface molecules. Considering a physisorbed water molecule at room temperature, the electronegativity of oxygen is higher than that of hydrogen. Thus, there are positive partial charges at the hydrogen atoms. The positive side of the physisorbed water dipoles attracts electrons to the surface of the semiconductor [7]. This leads to a bending down of the conduction and valence band at the surface. At higher temperatures, oxygen molecules react with the surface molecules and are reduced to oxygen ions that are chemisorbed to the semiconductor surface. Thus, electrons are accumulated at the surface, which results in an increase of the number of holes, leading to band bending at semiconductor surfaces. Water molecules, in the nearby area, are physisorbed to the semiconductor surface and replace the oxygen ion sites. The surface electrons depletion is neutralized. Hence, conductivity is increased. Semiconductor based humidity sensors are usually driven at temperatures around 200 °C because the signal change is higher and linear [17].

The third type of humidity-sensing material is an organic material being commonly a polymer. It is important to underline that polymers are an emerging candidate in many sensing applications due to low cost, commercial availability, and easy deposition on different transducers. Besides this, a photosensitive polymer adds advantages related to rapid polymerization, elimination of volatile organic solvents usage, and reduction of technological process steps. A polymeric layer in contact with water vapor shows a change in its conductivity or its dielectric constant ε. As water diffuses into a hydrophobic polymer layer like polyimide, poly(methyl methacrylate) (PMMA), cellulose acetate butyrate (CAB), or poly(ethyleneterephthalate) (PETT), the matrix absorbs water and only its relative permittivity ε undergoes changes [7]. Several studies demonstrate that hygroscopic polymer sensing layers present a good sensitivity to humidity at room temperature [14,18]. A linear sensing performance between 5% and 90% RH and a long-term stability has been shown due to the establishment of cross-linking and interpenetrating networks (IPN) [19]. 

Nowadays, capacitive humidity sensors dominate the market. Polymeric layers as a sensitive layer in combination with interdigitated electrodes are intensively studied [7]. Despite the availability of several low-cost relative humidity sensors on the market, new small and medium size companies are looking for mature technology for realizing RH sensors for a wide range of applications. Although polyimide films have been used as sensing layers for humidity sensors [18,20,21,22,23,24,25,26,27], complete reports about the properties of polyimide based capacitive for use as the sensing layer for RH are still missing. In the present work, we report detailed insights into the performance of a planar interdigitated transducer coated with polyimide sensing material having different thickness and morphologies. It is worth mentioning that planar IDTs take advantage only of the electric field lines penetrating the polymeric layer (in general the upper half of the transducer).

## 2. Experimental Details

A chemical sensor is a device that transforms chemical information, i.e., the analyte concentration, into an electrically useful signal. Basically, it consists of a transducer and a sensitive layer. The chemical information is obtained either due to a chemical reaction or due to a sorption process between the analyte and the sensitive layer. In our study, we fabricated capacitive humidity sensors, which monitor any changes of in the dielectric properties of the appropriate sensing material. The transducer was a pair of planar interdigitated electrodes. The sensing material was polymer-based material called polyimide.

### 2.1. Transducer Configuration

**Figure 1:**
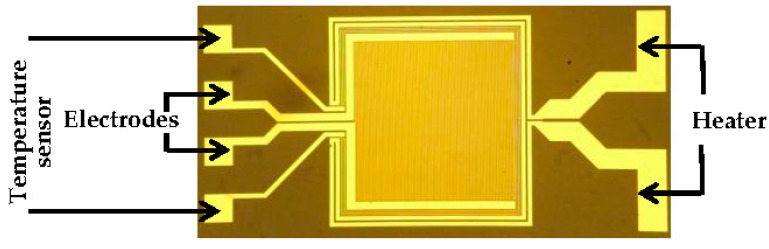
Layout of studied interdigitated transducer. The electrodes are surrounded by a temperature sensor and a heater.

A pair of interdigitated microelectrodes was produced by standard photolithography on a 620 µm thick glass substrate. For operating at elevated temperatures, quartz substrates are the best choice. After cleaning the glass substrate wafer, a thin adhesion layer of TiW (40 nm) followed by a thick gold layer (140 nm) were deposited by sputtering deposition technique. The electrodes were patterned by photolithography technique using the desired mask. The pattern included two wires around the interdigitated electrodes, one to heat the sensor and the other to measure the surrounding temperature (Figure 1). Each chip featured two interdigitated gold electrodes with filling area of 2 × 2 mm^2^. Finger width, length, and pitch were set to 6 µm, 2 mm, and 12 μm, resulting in 83 and 42 fingers, respectively.

### 2.2. Sensing Material Preparation

The polyimide used for humidity sensing layers was prepared from a photosensitive solvent developable polyamide acid ester. First, the substrate surface was cleaned with a stripper in an ultrasonic bath from any contaminants and organic impurities prior to the coating process in order to ensure a good adhesion of the sensing layer to the glass and metallic electrodes surface. The polyimide material was spin coated homogeneously on top of the planar electrodes on the 6-inch glass wafer. Different layer thicknesses were obtained, ranging from 1 µm to 11 µm. The spin coating parameters (spin speed and time) varied according to the desired final film thickness and the precursor initial viscosity. After spin coating, the films underwent a soft bake for a short period of time to drive out residual solvent. Then, the photosensitive layer was exposed to an appropriate dose using a mask for pad opening. The exposure dose was chosen based on the thickest layer in case of a variation of the coating thickness on the substrate. The wet development process was realized at room temperature for an optimal time and followed by flood exposure, without a mask, applied after developing to allow a complete crosslinking reaction. The imidization reaction took place between the amino group and the ester group resulting in an imine group. After completing the photolithographic process, the film was cured in a Yes Oven under N_2_ inert atmosphere. The curing temperature was 400 °C.

### 2.3. Transducer and Sensing Material

The coated wafer with polyimide sensing material was diced into 4.7 × 3.9 mm^2^ large individual chips. Each chip was mounted on an adapted printed circuit board (PCB), glued, and then gold wire bonded thereto. The PCB was equipped with zero insertion force (ZIF) connectors. 

### 2.4. Gas Mixing Apparatus

A reliable gas mixing apparatus, which is a part of a gas measurement setup, is an important system for characterizing gas sensors under desired gas mixtures. The dynamic volumetric method, described in reference [28], was adopted to generate a defined gas mixture in a reproducible way. For this study, the gas mixing apparatus was able to generate a constant level of absolute humidity by selecting the adopted ratio between the volumes of humid gas to the carrier gas, which is N_2_ or synthetic air.

### 2.5. Capacitance Measurements Using an Impedance Analyzer

**Figure 2:**
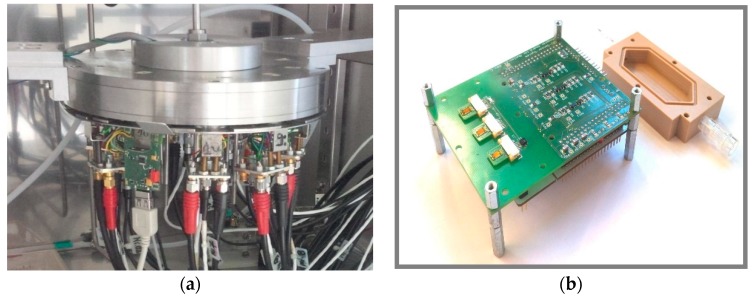
Photograph of (**a**) sensor chamber for the impedance analysis and (**b**) evaluation kit with a PEEK lid. Both can be connected to the gas mixing apparatus.

Up to 10 chips could be placed into a cylindrical stainless steel chamber (sensor chamber), which is connected to the gas mixing apparatus (Figure 2a). Consequently, they were simultaneously characterized under the same gas environment conditions. A radial distribution of the gas and isolating wall between the 10 chips ensured a continuous and controlled flow of the gas.

The electrical properties of the sensors were monitored by impedance spectroscopy using a Solartron gain-phase frequency analyzer model 1260 A controlled by a PC, permitting automated data collection. The impedance measurements could be carried out at different temperatures (from RT to 200 °C) by heating up the chip using the integrated heater around the electrodes. The measurements were performed from 1 MHz to 10 Hz to get the optimal frequency at which the sensor capacitance change linearly as a function of RH. Within another project, it is planned to develop a miniaturized system using the AD chip. The AC Voltage excitation of the AD chip is around 38 kHz. For this reason, most of the experimental measurements are done at 40 kHz.

For this study, the capacitance is recorded at 25 °C under different frequencies [29]. The sensor is subjected to a dynamic change of relative humidity levels and its capacitance is monitored. Most of the developed sensors capacitance were monitored under relative humidity levels ranging from 5% to 85% at 25 °C. The lower measured capacitance between 5% and 15% is considered as a base capacitance. The error bars of the capacitance are related to the impedance analyzer error.

### 2.6. Capacitance Measurements Using Evaluation Kit

An evaluation kit with high speed data acquisition was designed. Additional features are low power, low cost, compact size, and network compatibility. The used microcontroller STM32-L476RG possesses a low-power design, several Analog-to-Digital converters (ADC), and a touch sensing controller for capacitive measurements via the charge transfer method on board. The touch sensing controller consists of a number of internal switches. After loading the sensor capacitor with a constant voltage, the charge is transferred by the switches to an external reference capacitor (104 times bigger). This process is repeated N times, until a threshold voltage is reached on the reference capacitor. The capacitance of the sensor is then calculated by [30]

(1) $$C_{s}=-\frac{C_{REF}}{N}\ln\left(1-\frac{V_{th}}{V_{dd}}\right)$$

where:

*V_th_*: threshold voltage (1.5 V)
*V_dd_*: positive supply voltage (3.3 V)
*C_REF_*: reference capacitor
*N*: number of charge transfer cycles needed to charge the *C_s_*.

An extension circuit board was developed, which holds sensor connectors, heater driver circuit, resistance temperature detectors (RTD), and reference capacitors for the charge transfer method. Additionally, PT1000 and SHT25 were added as reference sensors and connectors for communication protocols (UART and I2C). The evaluation kit can simultaneously measure three sensors by measuring the capacitance and controlling the operating temperature by a proportional-integral-derivative (PID) algorithm. It permitted measuring the response and recovery times of the developed sensors by ensuring a short measurement interval and quick gas exchange ability. In order to use the evaluation kit together with the gas mixing apparatus, a PEEK lid was designed and screwed with a sealing ring onto the PCB, where three sensors sockets were positioned (Figure 2b). The sampling time was 150 ms. Therefore, the evaluation kit permitted measuring the response and recovery times of the developed sensors due to a short measurement interval and quick gas exchange ability.

## 3. Results and Discussion

### 3.1. FTIR Characterization

**Figure 3:**
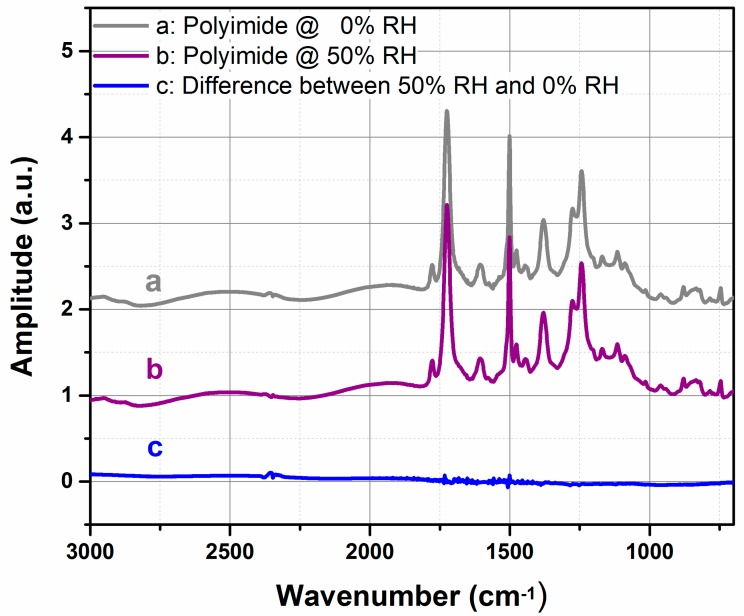
Two infrared spectra covering a range from 3000 and 800 cm^−1^ measured at zero RH (grey) and 50% RH (purple). The difference spectrum of both spectra is shown for clarity (blue). Between 3000 and 1800 cm^−1^, reflections on the flat polyimide surface cause interference. The small peaks at 2300 cm^−1^ result from remaining gaseous CO_2_ inside the FTIR chamber. RH, relative humidity.

Fourier-transform infrared spectroscopy (FTIR) is an extremely reliable method that provides chemical bond and molecular composition present of any partially transparent material. To realize FTIR spectroscopy for our purpose, silicon test wafer substrates (1 × 1 cm^2^) were coated with a polyimide layer and examined under IR radiation at different humidity levels by a FTIR spectrometer. A background reference curve is measured with an uncoated sample from the same silicon wafer. A first measurement is performed with a polyimide coated sample under dry condition inside the FTIR interior space at nearly 0% RH (grey curve in Figure 3). The same sample was then interconnected to a copper humidity chamber and placed in the FTIR beam path. The heatable copper chamber has a small water reservoir at the bottom. A thin tube, with a 1 mm diameter, ensures the connection between the chamber and the water reservoir. The chamber is equipped with two ZnS windows having strong IR transmitting properties. After closing the chamber, water molecules from the reservoir evaporate and diffuse through the thin tube into the chamber, where the sample coated with polyimide is placed. After 24 h, the relative humidity reached 50%. At this time, a second FTIR measurement is performed (purple curve). Both infrared spectra reveal a wave like interference pattern, which are visible especially in the range of 3000 to 1750 cm^−1^. Polymer-based sensors are usually based on the analytes diffusion into the polymer sensing matrix. FTIR spectra of a polyimide layer at 0% and 50% relative humidity reveal the absence of any chemical reaction between the polyimide layer and the water vapor molecules, which is infrared-sensitive in the range between 8000 and 800 cm^−1^. Therefore, chemisorption of water molecules with the polyimide matrix can be ruled out. The interaction of polyimide with water molecules is purely a physical absorption. Moreover, the FTIR spectra show the presence of several imide peaks, especially at about 1720 cm^−1^, 1780 cm^−1^, and at 1350 cm^−1^, which belong to C-N stretching vibration of the imide ring. This confirms that a successful imidization has been reached and finally an amorphous stable material is formed. 

### 3.2. Effect of Frequency Using Impedance Analyzer

**Figure 4:**
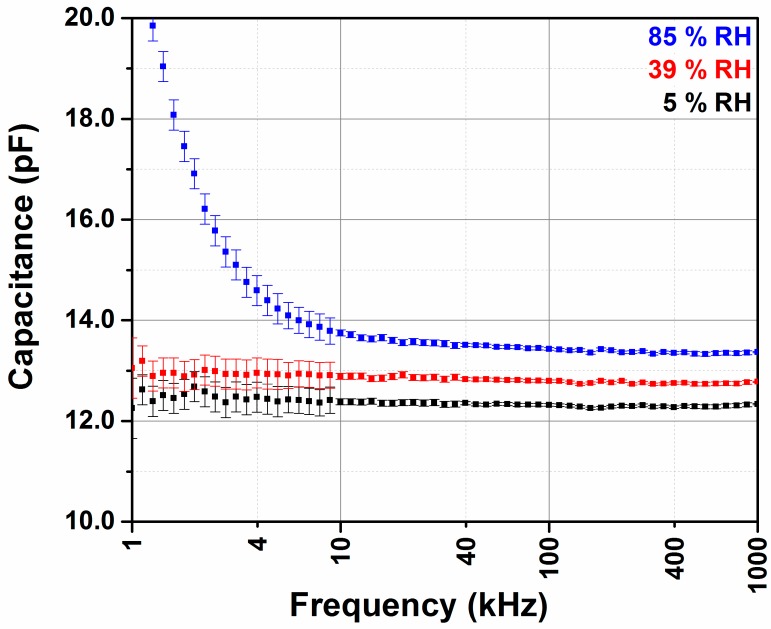
Frequency-dependent capacitance for different RH = 5% (black), 39% (red), and 85% (blue) of a 11 µm thick polyimide layer. The Error bars represent the uncertainty given by the impedance analyser.

The electrical properties of interdigitated transducer coated with polyimide, under different relative humidity levels, are monitored by impedance spectroscopy in the frequency range of 10 Hz to 1 MHz. The sensor chamber is situated inside a constant temperature incubator at 25 °C. Figure 4 represents the capacitance deduced from the complex impedance using resistor-capacitor (RC) parallel equivalent circuit versus the frequency for different relative humidity. Between 1 kHz and 1 MHz, polarization effects of water molecules do not contribute to the sensor’s capacitance and the measurement error is minimal. Thus, a linear behavior of the capacitance is obtained (Figure 4). The same constant trend of capacitance was also measured between 100 kHz and 10 MHz [24], where metallic interdigitated electrodes were printed on one side of a polyimide substrate. For extensive studies, 40 kHz was chosen as the measurement frequency for the impedance analysis. At 40 kHz, the error of the impedance analyzer is small. Additionally, the polarization of water molecules does not contribute to the impedance, as it is the case at high humidity levels and small frequencies.

### 3.3. Dynamic Mode Using the Impedance Analyzer

**Figure 5:**
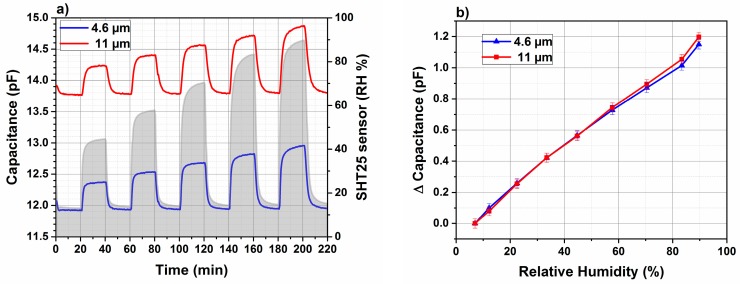
(**a**) Temporal evolution of capacitance of 4.6 µm and 11 µm polyimide layers at different RH measured by SHT25 commercial sensor. (**b**) Calibration curve of 4.6 (blue) and 11 µm (red) polyimide layers. The capacitance change is traced versus relative humidity at a frequency of 40 kHz.

The 4.6 and 11 μm thick polyimide films are deposited onto the IDTs. The capacitance is monitored as a function of time by an impedance analyzer at 40 kHz frequency under different RH levels. Figure 5a displays the typical response and recovery curves of the as-fabricated humidity sensor to different amount of relative humidity in the carrier gas. The sensors are alternatively exposed to different RH, which are varied from 16% to 85%. The exposure time of the sensor to the RH, higher than background value 16%, is fixed to 20 min. Once the RH is decreased to the background value 16%, the sensors capacitance returns to the initial base capacitance in a reversible without observing any pronounced hysteresis. 

Figure 5b shows the capacitance reached after exposing the sensors to different RH for 20 min. The capacitance versus different RH values increases monotonically by increasing the RH from 10% to 80% for polyimide layers with two different thicknesses. The RH is also determined using commercial SHT25 sensor. Both developed humidity sensors represent a good linearity with respect to variations in relative humidity. It is also observed that the capacitance changes of IDTs coated with two different polyimide thicknesses 4.6 µm and 11 µm are identical. As the capacitance change results from a bulk absorption of water molecules a decrease of the capacitance change would have been expected as the polyimide thickness decreases. Therefore, a complementary study is necessary to investigate the penetration depth of the electrical field lines by the mean of FEM software to explain the obtained calibration curves for different polyimide thicknesses.

Above 80% and below 15%, a deviation from the linear behavior should be expected. In addition, the hysteresis characteristic of the polyimide-based humidity sensor was obtained in static mode. The RH was increased continuously from 12% to 85% by a step of 10%. Then, RH has to be decreased from 85% to 12% by a step of 10%. SHT25 gave the reference measurement of the humidity. The hysteresis is evaluated to be less than 2%.

### 3.4. Penetration Depth Using FEM Simulation

COMSOL Multiphysics (version 4.3a) is a simulation software, which applies finite element simulation to a 3D model of the structure to be studied in this work. The CAD is used to build a 3D model that consists of several layers: glass substrate, gold layer, sensitive layer (optional), and air layer. The derived capacitance of a bare IDT by FEM simulations agree well with the one measured by the impedance analyzer. By combining simulations with different dielectric constants of the sensitive layer and the measured sensitivity of the coated IDT, the correlation between the dielectric constant of the polyimide layer and the relative humidity was determined. Therefore, at 25% RH the dielectric constant of polyimide is equal to 3.5.

**Figure 6:**
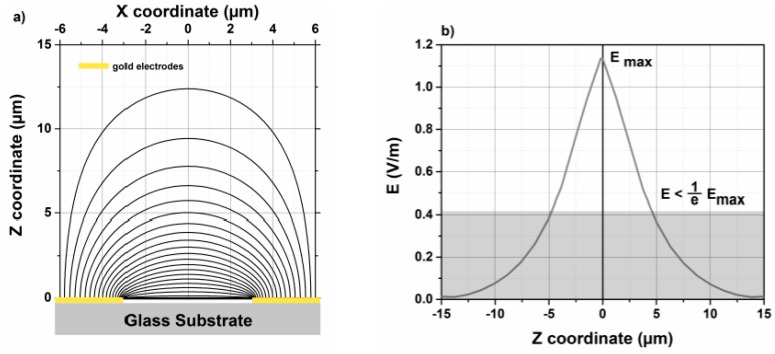
(**a**) Stream lines distribution between two adjacent electrodes on glass a substrate. The gold electrode width and the gap between two adjacent electrodes are fixed to 6 µm. (**b**) Electric field strength along a perpendicular axis to the IDT’s planar surface and situated in the middle between two electrodes adjacent electrodes.

Calculation of the penetration depth is simulated to determine the maximum reasonable polyimide layer thickness. Figure 6a shows stream lines of the electric field for used IDTs having a space width between consecutive electrodes of 6 µm and an electrode width of 6 µm. The simulated structure is identical to the one used as a transducer for the developed humidity sensor. Stream lines of the electric field leave the electrode perpendicular to the surface. The electric field lines form ellipsoids going from positive to negative electrodes. As a consequence, IDTs are coated with a polyimide sensitive layer. The 3D models for the FEM simulations consist of a 4.6 μm thick polyimide layer on the top of IDTs. The dielectric constant of polyimide is higher than that of air (ε_PI_~3.5). The values of the penetration depth are obtained from COMSOL plots of the electric field. Figure 6b shows a plot of the electric field strength along a perpendicular axis to the IDT’s planar surface and situated in the middle between two adjacent electrodes. The maximum of the electric field strength is reached at z = −0.1 μm, which is located in the middle of the gold electrodes. Negative z values represent the positions inside the glass substrate and positive z values those inside the sensing layer. The electric field tends to zero if the z values tend to infinity. The penetration depth is defined as the depth at which the electric field strength falls below E/e~0.37 E (grey area). The graph, in Figure 5b, represents an IDT with 6 μm electrode space and 4.6 μm polyimide as sensitive layer. The penetration depth inside the polyimide layer is 4.56 μm. The penetration depth value is not significantly influenced by the electrode finger width. However, it is correlated to the gap between the electrodes. In this case, the area above 4.6 µm beyond the transducer surface does not contribute to the capacitance significantly. That is why the sensitivity of a 4.6 µm thick polyimide layer and a 11 µm layer are equal (as described in the previous Section 3.3). Generally, the sensing mechanism of the used material determines the ideal IDT structures. Smaller structures are better suited for thin layers.

### 3.5. Nanograss Polyimide Film

In the last years, high aspect ratio structures from nature have inspired engineering and scientists to develop new materials. The nanostructures, having superhydrophobic surface properties, is still attracting considerable attention for application in photovoltaic [31], biosensors [32], and power source [33]. Simple methods to modify the polymeric layer structure have been developed, leading to superhydrophobic surfaces (water-repellent surfaces), without inducing a significant change in the chemistry of the polymeric layer.

In the case of polyimide material, Lee et al. proposed that etching of the layer at pressure equal to 100 mTorr for 5 min results in a nanograss structure [20]. A pressure of 720 mTorr was commonly used to obtain a more homogeneous surface feature [34]. This nanograss film showed a very small response time to moisture.

**Figure 7:**
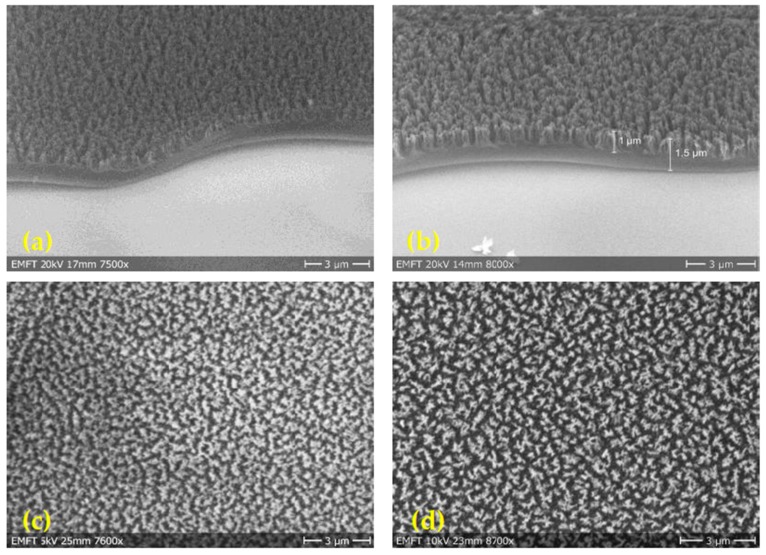
SEM cross-section pictures of plasma etched polyimide layers using different pressures: (**a**) p = 720 mTorr and (**b**) p = 50 mTorr. The thickness of the resulted grass structure is indicated by arrows. SEM top view pictures of etched polyimide layers at (**c**) p = 720 mTorr resulting in a roughened surface with a surface coverage compared to (**d**) p = 50 mTorr.

In the present work, the plasma etching process of polyimide films is carried out under oxygen plasma at two different pressures, 50 mTorr and 720 mTorr, using a reactive ion etching chamber from Plasmalab. Oxygen plasma is generated by a radio frequency (RF) power source. The RF power for both working pressures is fixed to 113 W. The bias voltage, which built up between oxygen source and sample chuck, is 380 V and 140 V for lower and higher etching pressure, respectively. The bias voltage reflects the kinetic energy of the etching reactants. Hence, the oxygen ions with the lower pressure hit the polyimide surface with a higher kinetic energy. The etching rate of polyimide layer is determined by measuring the layer thickness by a profilometer after an etching time of 2 min. The plasma etched samples are analyzed by SEM, as presented in Figure 7. The thickness of both films is nearly equal. Both etching processes increase surface roughness and change the surface morphology of the polyimide layer. At the edge of the layer, both samples look like nanograss structures but the orientation of the fibers is different. In the inner area, both etched films consist of two layers. The bottom layer is a bulk polyimide layer that has not been affected by the etching process and the upper layer is a fiber-like layer. In case of the low pressure etched film, the nanograss covers 67% of the whole film thickness. In contrast, only 42% of the whole film thickness is structured in case of a higher-pressure plasma etching process. 

Previous theoretical work established a correlation between pressure and etching rate [35] to explain the observation reported by SEM results. At high pressure, the number of collision events between oxygen ions inside the plasma increases drastically. Thus, the kinetic energy of the plasma ions decreases, which lowers the etching rate. At low pressures, the number of oxygen ions in the plasma is lower. Therefore, the number of reactants hitting the substrate surface decreases and the etch rate is decreased. As the number of collision between oxygen atoms during process is reduced, the reactants hit perpendicularly the polymer surface. For this reason, the fibers formed by the low pressure etching process are oriented perpendicularly to the substrate surface, more like grass. Contrary to the high pressure etching process, the fibers formed in the polymer layer are disordered, because the angles of incidence of the etching reactants are statistically distributed.

The realized etching process under oxygen plasma at low pressures, 50 mTorr, yields nanograss-like polyimide structures with higher etching rate [20]. In this study, the etching process results vertically oriented nanograss structures with 1 µm height on the top of 0.5 µm flat polyimide layer on the substrate. However, the dimensions of the individual fibers of the nanograss are comparable, around 50 nm in diameter with an interspacing distance of around 100 nm.

### 3.6. Humidity Sensor Performance

A series of photosensitive polyimide layers are deposited onto IDTs. The layers present different thickness and surface morphologies. To characterize the performance of chemical sensors under development, sensors are placed into a hermitic test measurement chamber and the sensors capacitance is monitored under different humidity levels by an impedance analyzer. A commercial humidity sensor, SHT25 device with a standard calibration, is selected for reference.

#### 3.6.1. Dynamic Response Using Impedance Analyzer

**Figure 8:**
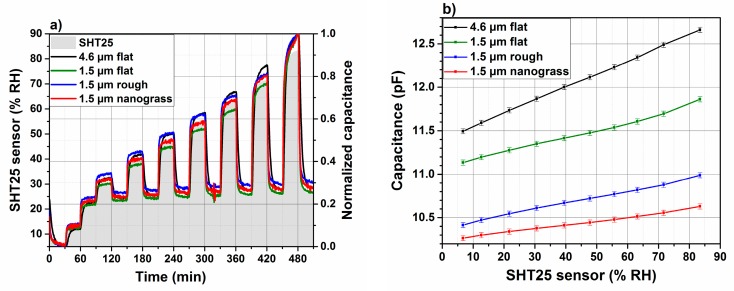
(**a**) Normalized capacitance of humidity sensors measured at 40 kHz versus time under different RH levels and for different thicknesses and surface morphologies. (**b**) Calibration curves of developed humidity sensors with different thicknesses and surface morphologies as indicated.

Figure 8a shows the measured capacitance with the impedance analyzer inside the sensor chamber, which is normalized versus time for polyimide material with different thickness and surface morphologies under different RH levels. The signal delivered by a commercial reference sensor, SHT25, operated under the same conditions, is superposed to the developed sensors data. The Figure 8b shows the capacitance of the measured sensors in Figure 7a, as a function of relative humidity measured by SHT25. The capacitance refers to the measured value after 30 min in a certain humidity. For all sensors, the calibration curves present a linear behaviour. In Table 1, sensitivity and linearity of developed sensors are summarized. The sensitivity is expresed as the ratio of capacitance to relative humidity:

(2) $$Sensitivity\;\left(fF\;/\;\%\;RH\right)=\frac{C_{RH=85\%}-C_{RH=6\%}}{RH_{85\%}-RH_{6\%}}$$

where: C_RH=85%_ and C_RH=6%_ are the measured capacitance at high and low level of relative humidity, respectively.

The sensitivity of the sensors decreases from 15 to 9.5 fF/% RH by decreasing the polyimide thickness from 4.6 to 1.5 µm with a flat morphology. As described in Section 3.4, the sensitive region of the transducer is situated at 4.6 µm beyond the transducers surface. Obviously, by decreasing the polyimide layer thickness, the sensitivity also decreases. Increasing the roughness of a 1.5 µm polyimide leads to a decrease in the sensor sensitivity. However, the calibration curve linearity is not affected. However, it was noted that a polyimide-based humidity sensor presents an increase in the sensitivity from 351 to 506 fF/% RH if the the layer thickness is etched from 400 to 100 nm under O2 plasma [27]. This improvement in the sensitivity is related to the designed electrodes, which are not planar but parallel. The bottom electrode is deposited in a substrate cavity and the top electrode has a comb shape. The polyimide layer is sandwiched between the bottom and the top electrodes. Such parallel electrodes configuration is often related to high sensitivity [26,27].

#### 3.6.2. Response and Recovery Times Using Evaluation Kit

Although a gas measurement setup, combining the gas mixing apparatus and electrical measurements using impedance analyzer, is a standard method to characterize the performance of chemical sensors under development, the response and recovery times, typically less than 3 min, are difficult to be accurately monitored for RH sensors under test. The reason is that some minutes (5 min) are needed to ensure a complete gas exchange inside the testing chamber [28]. To overcome the issue of studying the response and recovery times of the developed humidity sensors, a miniaturized evaluation kit is developed as a complementary setup. This evaluation kit is considered as a complementary tool to monitor simultaneously the electrical characteristic of the sensors and to measure their response and recovery times. Moreover, it can also be used to examine the sensor’s performance in more realistic conditions and to study its long-term stability. The gas mixing apparatus is used to provide a humid test gas. A second gas flow of dry carrier gas was provided by a separate mass flow controller. A Luer Lock vent is used to switch between the humid and the dry test gas. Hence, the gas exchange time and the measurement interval are fixed to 400 ms and 150 ms, respectively.

**Figure 9:**
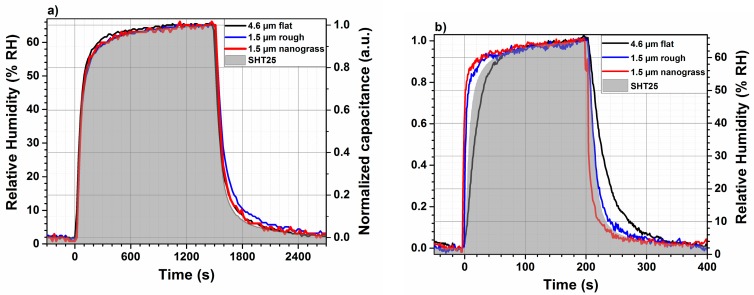
Response and recovery times of relative humidity sensor compared to a commercial one (SHT25) measured inside the chamber of the (**a**) gas mixing setup at 40 kHz (**b**) evaluation kit.

The response and recovery times in this study are defined as the times required for the sensor capacitance to reach 90% of the maximum measured signal, when the humidity is changed between two humidity levels (here from 6% RH to 67%). Figure 9 summarizes the response and recovery times of three different developed humidity sensors having different polyimide film thicknesses (11 µm and 1.5 µm) and morphologies (flat, rough, and nanograss). It is worth noting that 1.5 µm flat polyimide sensors have been excluded from further investigations, because their response and recovery times are higher as compared to the rough film with the same thickness.

As the sensing mechanism is based on diffusion, the thinner sensing layer shows a smaller response as well as a smaller recovery time. However, the nanograss-like layer shows the best performance even in comparison to commercial humidity sensors. An optimization of the transducer design and its miniaturization will lead to a further improved sensor performance. The measured capacitance, normalized, of the polyimide-based RH sensor (for both thicknesses and morphologies) as a function of time is shown in Figure 9. The relative humidity is determined by a commercial humidity sensor SHT25, placed in the same humidity condition as the developed ones.

The RH level in the test chamber is varied from dry to 65% and back to the initial level 6% after some seconds. The sensor samples with a polyimide thickness of 4.6 µm having a flat morphology and 1.5 µm rough surface and 1.5 µm nanograss-like surface present the same response and recovery times once measured in the chamber of gas mixing setup. As mentioned in Section 2, the evaluation kit is more appropriate to deduce response and recovery times of sensors. Figure 9b reports the response time and recovery times of the prepared samples with different thickness and morphologies. The sensors are placed into the chamber of the evaluation kit, which is connected to the gas mixing unit. The 1.5 µm nanograss polyimide shows the lowest response time of about 18 s in comparison to the same thickness of rough polyimide layer (1.5 µm rough) and 4.6 µm flat layer and the commercial sensor; see Table 2. Regarding the response time, the same observation is valid for nanograss film. This means that the recovery time of a nanograss film is 31 s, which is lower than 1.5 µm rough polyimide layer (45 s), 4.6 µm flat layer (80 s), and the commercial sensor (40 s). The extremely rapid response and recovery times for the sensing layer having a rough and nanograss morphology is probably related to the porous structure in comparison to the flat morphology. Such porous structure of the sensing layer, created during the plasma etch process, permits a rapid diffusion of water molecule inside and outside the matrix. Besides this, the layer thickness is another important parameter, which should be optimized for the chosen interdigitated electrodes. The same observation regarding the response and recovery times were noted for different polyimide (Kapton HN50) thicknesses [25]. The response time was decreases from 25 min to 4.75 min by decreasing the Kapton HN50 thickness respectively from 25 µm to 7.5 µm. In our case of polyimide-based humidity sensors, an increase in the recovery time in comparison to a response time of 50% to 70% was obtained, which is higher than the calculated value 26% from other work [25]. However, the commercial sensor shows the same response and recovery times. 

It is worth noting that the sensor responses are uncorrelated to the used ambient air. No significant differences are detected of measuring the sensors response under carrier gases N_2_ or synthetic air.

Generally, the adsorption and desorption of a specific analyte carried by an inert gas into a sensing material matrix is governed by either diffusion process or thermodynamically controlled process or even both processes. In case of an ideal diffusion-controlled process, the response time and the recovery time should be equal. In the thermodynamically controlled process, the adsorption is an exothermic process, whereas desorption is an endothermic process. Consequently, desorption requires higher external energy which is reflected by a higher recovery time. An adsorbed molecule on the surface of a sensing layer at certain temperature remains stable at this state. A moderate increase of the sensor temperature, in the absence of any interaction between the sensing material and the analyte, leads to desorption of the desorbed molecule from the surface and returns into its initial gas form.

As confirmed from the FTIR measurements, the polyimide material presents no chemical interaction between the polymer backbone and water molecules. However, the polyimide layer with rough and nanograss-like morphologies show a large difference between their response and recovery times (72%) to humidity. This large difference between the two parameters describing the humidity sensor is probably due to structure changes into the polyimide matrix induced by the plasma process. These structure changes in the polymer induced by the O_2_ plasma etch will be subject of future studies.

## 4. Conclusions

In summary, a systematic examination of the sensor performance of a series of polyimide layers with different thickness and morphologies (flat, etched, and nanograss) exposed to different humidity levels are measured simultaneously in the same test chambers. We have successfully demonstrated that the nanostructure of polyimide to obtain nanograss can be well controlled by adjustment of a series of O_2_ plasma etch parameters. The nanograss morphology exhibits the best performance among the studied morphology types. The ideal polyimide layer thickness turns out to be equal to the penetration depth of the used interdigitated transducer. The sensor signal, capacitance, follows a linear dependence with RH. Polyimide sensing layers with nanograss morphology ensure the best humidity sensor performance regarding the response and recovery times. The optimal polyimide layer thickness and morphology can be chosen as a function of the envisaged application of relative humidity sensors and the used interdigitated transducer.

## Figures and Tables

**Table 1:** Sensitivity and linearity of the developed polyimide-based humidity sensor with different morphologies at 40 kHz and 25 °C. The error of the impedance analyzer is 0.3 pF.

	4.6 µm Flat	1.5 µm Flat	1.5 µm Rough	1.5 µm Nanograss
Sensitivity (fF/% RH)	15.2	9.48	7.47	4.75
linearity	0.999	0.995	0.998	0.998

**Table 2:** Response and recovery times of polyimide-based humidity sensor with different morphologies. The error bar is 2% due to reference capacitor and analog-digital converter (ADC).

	RH Sensor (SHT 25)	4.6 µm Flat	1.5 µm Rough	1.5 µm Nanograss
Response time τ90_res_ (s)	42	54	26	18
Recovery time τ90_rec_ (s)	40	80	45	31
Increase in τ90_rec_ compared to τ90_res_ $\left(\frac{\tau_{rec}}{\tau_{res}}-1\right)*100\%$	−5	48	73	72
